# Phase 2 open-label extention (OLE) study of patisiran, an investigational siRNA agent for familial amyloidotic polyneuropathy (FAP)

**DOI:** 10.1186/1750-1172-10-S1-O20

**Published:** 2015-11-02

**Authors:** David Adams, Ole B Suhr, Isabel Conceicao, Marcia Waddington-Cruz, Hartmut Schmidt, Juan Buades, Josep Campistol, Teresa Coehlo

**Affiliations:** 1 CHU Bicêtre, APHP, French Reference Centre For FAP (NNERF), 94275, LE KREMLIN-BICETRE, France; 2 Umea University, Department of Public Health and Clinical Medicine, SE-901 85, Umea, Sweden; 3 Centro Hospitalar Lisboa Norte-Hospital de Santa Maria, Department of Neurology, 1649-028, Lisbon, Portugal; 4 Hospital Universitario Clementino Fraga Filho, UFRJ, CEP21941-913, Rio de Janeiro, Brazil; 5 The University Hospital of Münster, Department of Transplantation, 48149, Münster, Germany; 6 Hospital Son Llatzer, Servicio de Medicina Interna, 7198, Palma de Mallorca, Spain; 7 Hospital Clinic Barcelona, Instituto Clinic de Nefrologia y Urologia (ICNU), 8036, Barcelona, Spain; 8 Hospital de Santo Antonio, Unidade Clinica de Paramiloidose, 4099-001, Porto, Portugal

## Background

Familial amyloidotic polyneuropathy (FAP) is a progressive and fatal, autosomal dominant disease caused by deposition of mutant and wild-type transthyretin (TTR). Patisiran is an investigational, systemically administered lipid nanoparticle (LNP) formulation of a small interfering RNA (siRNA) targeting wild-type and mutant TTR. This formulation delivers the siRNA predominantly to the liver, thereby inhibiting synthesis of TTR at the primary site of production. A recently completed multi-center, multi-dose Phase 2 trial of patisiran in FAP patients (N=29) showed >80% sustained mean knockdown of serum TTR when administered at a dose of 0.3 mg/kg every 3 weeks with a generally favorable safety profile (Suhr O, ISA 2014).

## Methods

A Phase 2 open-label extension (OLE) study of patisiran in patients with FAP who participated in the aforementioned trial, was initiated in October 2013. The primary objective of the study is to evaluate the safety and tolerability of 0.3 mg/kg patisiran administered intravenously once every 3 weeks for up to 2 years. Secondary objectives include assessment of patisiran's effect on serum TTR levels, as well as evaluation every 6 months of its impact on clinical measures, including the mNIS+7 composite neurologic impairment score and quality of life (QOL).

## Results

Twenty-seven patients were enrolled; median age 64 years (range: 29-77 years). Chronic dosing with patisiran has been generally well tolerated. Three patients experienced serious adverse events unrelated to study drug. Flushing and infusion-related reactions were observed in 22.2% and 18.5% of the patients, respectively; these were mild in severity, and did not result in any discontinuations. Sustained mean serum TTR lowering of approximately 80% was achieved, with further mean nadir of up to 88% between doses for approximately 16 months. Stabilization of quality of life (QOL) measures was observed. Among the 20 evaluable patients at the time of data cutoff, neuropathy impairment scores were stable through 12 months with a mean change in mNIS+7 and NIS of -2.5 and 0.4 points, respectively; this compares favorably to the 10-18 point increase in neurologic impairment scores estimated at 12 months from prior FAP studies in a patient population with similar baseline NIS.

## Conclusion

Data from this Phase 2 OLE study demonstrate that 12-months of patisiran administration was well-tolerated, resulted in sustained mean serum TTR lowering, and has the potential to halt neuropathy progression. As of March 2015, dosing continues for all patients; 18-month results will be presented.

